# Topics of the Month

**Published:** 1892-09

**Authors:** 


### BUFFALO MEDICAL AND SURGICAL JOURNAL

A MONTHLY REVIEW OF MEDICINE AND SURGERY.

*EDITORS:*

THOMAS LOTHROP, M. D. - - WM. WARREN POTTER, M. D.

All communications, whether of a literary or business character, should be addressed to the managing editor: 284 FRANKLIN STREET, BUFFALO, N. Y.

VOL. XXXII. SEPTEMBER, 1892. No. 2.

### TOPICS OF THE MONTH.

DR. IRVING A. WATSON, of Concord, N. H., Secretary of the American Public Health Association, contributes an article to the August, 1892, *Sanitarian*, entitled, The Republic of Mexico, Medicine, Curative and Preventive. This paper was read before the New Hampshire Medical Society, at Concord, June 20, 1892, and is a review of the medical status in that interesting republic. It deserves to be read by every friend of improved medical education, and should be circulated widely as a reprint. It is interesting to observe the advanced standard that prevails in Mexico, which compares with our own country to our infinite discredit.

Dr. Watson says :

> The requirements for the degree of M. D., at the School of Medicine, in the City of Mexico, are five years of study in the preparatory schools, and five years at the medical school, and even then a degree is not granted unless the student passes in the required examinations, which are in themselves rigid and thorough. The law of the country prohibits any person who does not possess the degree of M. D., from practising in any place where there is a legally qualified physician. In other words, when a graduate in the study of medicine locates in a town, all persons in the practice of medicine in that place not equally well qualified are obliged to cease the practice of medicine there.

And yet in the Empire State, in the year of our Lord 1892, we find medical students, medical professors, politicians, quacks, and what not, all combined to nullify the provisions of a law that would ask far less than is required of the graduate of medicine in the Republic of Mexico.

---

THE Illinois State Board of Health has issued an abstract of the minutes of the quarterly meeting, held at Chicago, July 27, 1892.

As is well known, this Board of Health has control of the practice of medicine in Illinois, in addition to its other duties. It is a pioneer in advancing the standard of medicine in this country, and its reports are always examined with great interest. In the abstract before us, we find a classification of the medical colleges of the United States into three groups:

1. Colleges requiring four or more years of study and four or more terms of lectures as conditions of graduation.

2. Colleges requiring four or more years of study and three terms of lectures as conditions of graduation.

3. Colleges requiring three or more years of study with three or more terms of lectures as conditions of graduations.

We take pleasure in printing the names of the medical schools of the first class, which are as follows:

Chicago Medical College, Medical School of Northwestern University, Chicago, Ill.; Harvard University Medical School, Boston, Mass.; Boston University School of Medicine, Boston, Mass.; Department of Medicine and Surgery, University of Michigan, Ann Arbor, Mich.; Leonard Medical School, Raleigh, N. C.; Medical University, Faculty of Medicine, Montreal, Que.; University of Toronto, Faculty of Medicine, Toronto, Ont.; Ecole de Medecine et de Chirurgie, Montreal, Que.; Trinity Medical College, Toronto, Ont.; Laval University, Medical Departments, Quebec and Montreal, Que.; Royal College of Physicians and Surgeons, Kingston, Ont.; Halifax Medical College, Halifax, N. S.; Dalhousie University, Faculty of Medicine, Halifax, N. S.; University of Bishop's College, Faculty of Medicine, Montreal, Que.; Medical Department of Western University, London, Ont.; Woman's Medical College, Toronto, Ont.; Women's Medical College, Kingston, Ont.; Manitoba Medical College, Winnipeg, Man.

We hope it will be soon required in the State of New York to place their colleges on this honor roll.

---

THE Necessity and Best Method of Regulating the Practice of Medicine, is the title of an address delivered by Dr. Perry H. Millard before the American Academy of Medicine, at it annual meeting, June 6, 1892. This is a plea for an advanced standard in medical education, and is one of the most interesting papers on the subject that we have lately read.

In the course of the discussion, Dr. Millard pleads for adequate medical legislation throughout the United States, looking to the establishment of separate State Examining and Licensing Boards. He submits the following statistics in support of his argument, which deserve to be preserved in the memory of every American physician who is interested in the subject of medical pedagogics and a separate State license. Ratio of physicians to population: Sweden, 1 to 7,000; Italy, 1 to 3,500; Germany, 1 to 3,000; Austria-Hungary, 1 to 2,400; France, 1 to 2,000; United States, 1 to 600.

Dr. Millard remarks that Minnesota has a smaller number of physicians to its inhabitants than any State in the Union, and St. Paul a smaller number to its inhabitants than any of the larger cities of the United States. He believes the result in that city is wholly due to efficient legislation; and that the effect of the present law has been to enhance the welfare of both the profession and the public.

---

In a letter to the *Times and Register*, published in its issue of July 23, 1892, Dr. Joseph Price, of Philadelphia, pays a deserved tribute to Dr. Alice Bennett, who has had the management of the Woman's Department in the Norristown Insane Asylum since its erection, twelve years ago.

It is not long since it was regarded as an experiment to employ women physicians in hospitals for the insane. The principle has now become established as one, if not of necessity, at least of propriety, of which Dr. Alice Bennett's success is ample testimony.

Dr. Price says:

> This remarkable woman has placed the management of this institution upon a high plane. We doubt whether there exists an institution of the kind in our own or any other country under more perfect management. It is a model of tasteful, orderly appointment and cleanliness, and that quiet which would not be expected in an institution for the insane.

We wish we had space to give the letter entire, for it is worthy of wide circulation. Dr. Price concludes as follows:

> It is a matter of high congratulation that the almost perfect management of the Norristown institution is that of a woman. The stimulus of the responsibility by such positions is the highest of educational influences, and women should push into these kindred places of trust wherever there is an open door.

---

At a meeting of the Michigan State Board of Health, held July 12, 1892, the Secretary, Dr. H. B. Baker, called attention to the reported presence of cholera in foreign countries, and the possibility of its being brought to our shores. Dr. Baker remarked that it would be a particularly unfortunate time if cholera should reach Chicago or Detroit during the period of the World's Fair. The Michigan State Board of Health is prepared to instruct local health officers relating to the restriction of cholera, and its action is most timely in reference to its possible invasion. Great maritime ports like New York are always the most dangerous avenues of approach, and we have no doubt our own State Board of Health, together with the National authorities, will furnish all the protection that preventive science and skill can command.

In our own city the health department is under capable medical administration, and we have every reason to believe that all avenues of approach will be as carefully guarded as possible, provided our municipal authorities make timely appropriation of money to arm the Health Commissioner and his assistants with the sinews of prevention. Fifty thousand dollars should be placed at the disposal of Health Commissioner Wende at once, to be used, if necessary, and at his discretion, for the prevention and restriction of infectious epidemics.

---

The Sixteenth Year Book, containing the annual report of the Board of Managers of the New York State Reformatory, at Elmira, for the year ending September, 1891, contains valuable information bearing on the question of the methods of reform as related to the refractory youths of this State. Dr. William C. Wey is President of the Board of Managers of this excellent institution, and to his sagacious and able management the institution owes a large measure of its success. It is, strictly speaking, an educational and physical training school, dealing with moral, physical, and sanitary questions as a broad and underlying basis. The report is well illustrated with several phototypes, which add much to the interest of the brochure.

---

The New York *Medical Times* has so concisely stated the relationship existing between publisher and subscriber in so far as medical journals are concerned, that we cannot resist the temptation to print the following :

> It is the custom of nearly all medical journals to continue subscriptions until notified that the journal is no longer wanted, or it becomes apparent to the publisher that there is no intention of the subscriber ever making any pecuniary return. The law holds a subscriber responsible for a periodical so long as it is taken from the office, and yet a medical journal very seldom avails itself of this very wise legal provision. Every year a bill is sent to each subscriber with the amount of indebtedness. It is certainly not too much to ask if the journal is wanted, a prompt return of the subscription, and if it is not wanted, a postal asking for its discontinuance. A second notice from a subscriber to discontinue will never be needed. The publisher of the *Medical Times*, while always glad to extend its circulation, has no wish to have that circulation include a single person who does not find something of interest in its pages. The conducting of a medical journal is in no sense a money-making business, more especially when it is entirely independent of great publishing or drug houses, to whom it may be important as an advertising medium. Again the publisher assures its readers that while he warmly appreciates every effort in the past of the friends of the *Times* to extend its circulation, any notice to discontinue will be promptly heeded.

The above so nearly expresses our attitude and belief in this matter, that we respectfully invite attention to it. The present management of the JOURNAL has always refrained from introducing any business question before its subscribers in its editorial columns, but we trust, in this instance, we will be pardoned for so doing, since it is more of an explanation of our relations with our subscribers than a disposition to complain or criticise.

---

AT THE Convention of the National Association of Railway Surgeons, held at Old Point Comfort, in May, 1892, Dr. Joshua Chitwood, of Connersville, Ind., read a paper entitled, Cranial Injuries, with a Report of a Case. The trend of the paper was to advise what he is pleased to call "conservatism" in the management of head injuries in general, where the symptoms are obscure, or the injury is not sufficiently plain in its external manifestation to justify surgical interference. In the discussion of this paper, Dr. Emery Lanphear, of Kansas City, speaks as follows :

I enter a solemn protest against the so-called conservative treatment of injuries of the head. There is no injury that is so important to the railway surgeon as an injury involving the bone of the cranium. There is no injury that is so poorly treated, as a rule.

In regard to the assertion of the essayist, that injuries which will perhaps result in epilepsy eventually should be left until the epileptic symptoms make their appearance, I do not believe it to be based upon rational surgery, and I would cite the assertion of the late Professor Agnew in the last paper which he prepared, in which he stated not only in ordinary print, but in italics, that every case of injury of the head, which would eventually terminate in an epilepsy, should be operated upon directly after the reception of the injury, if possible ; because experience has taught those who have operated for traumatic epilepsy that very few cases terminate favorably if left until after the epileptic manifestations have appeared ; whereas, injuries which would necessarily result because of the injury to the motor area in epilepsy, if operated on at the time of the accident, or directly thereafter, may be prevented in a large proportion of cases.

Again, we go too far in letting our patients alone when they are unconscious. From an experience of over forty cases of brain surgery, I have learned that every case of injury to the head, in which the comatose condition persists for more than two or three hours, demands imperatively that the surgeon should go in and ascertain what the injury is. If he enters the cranium with clean hands and clean instruments, there is absolutely no danger to be feared from his operative interference ; whereas, if he does not investigate the case, he may find too late that he has had rupture of the middle meningeal artery with the formation of a clot which will produce death, or, at least, very serious symptoms, for which the company he represents will be liable. I, therefore, repeat that we can carry conservative treatment entirely too far. I, for one, enter a hearty plea for more invasion of the cranial cavity and less conservatism in those cases accompanied by coma. [Applause.]

The remarks of Dr. Lanphear really cover the whole question so pointedly that we have quoted them in full. There is a tendency just now in many quarters to demand so-called "conservative" surgery for grave and destructive conditions, either from disease or injury, which relate to the several cavities of the body. In our review of the Southern Surgical and Gynecological Transactions in the August number of the JOURNAL, we took occasion to refer to this subject as related to the cavities of the pelvis and abdomen. Following out this line of thought, we have called attention to it as pertaining to the cranial cavity, and we invite the attention of our readers to a careful and considerate study of this side of the question.

---

THE *Ontario Medical Journal* was announced to be published in Toronto, and the first number to appear on or about the 15th of August, 1892. It is the organ of the College of Physicians and Surgeons, of Ontario, and is supplied, by order of the Council, to to every medical practitioner in Ontario, free.

Dr. R. B. Orr is announced as managing editor, whose address is 147 Cowan avenue, Toronto, Canada.

BULLETIN No. 3 of the Harvard Medical School Association, containing a report of the second annual meeting, held in Boston, June 28, 1892, is received. The President of this Association is Dr. James R. Chadwick, of Boston, and the Secretary is Dr. Robert Williamson Lovett, also of Boston. This bulletin contains the proceedings of the annual meeting, together with the speeches made at the dinner. It is one of the handsomest alumni publications that we have ever seen, and ought to serve as a model for these organizations in other medical schools.

---

THE *Weekly Bulletin of Newspaper and Periodical Literature*, published at 5 Somerset street, Boston, Mass., is one of the most useful catalogues of events that we are familiar with. It contains, besides all the current weekly news, a department of classified medical literature, including references to articles from the BUFFALO MEDICAL AND SURGICAL JOURNAL. Its value to readers, writers, and students is sufficiently indicated by its title, and it ought to be on the library table of every author, teacher, and student in letters, law, medicine, and science.

